# Recurrent eosinophilic pleuritis caused by sparganum infection: A case report and review of the literature

**DOI:** 10.1097/MD.0000000000020226

**Published:** 2020-05-29

**Authors:** Liangjie Fang, Yuehong Wang, Qiqi Gao, Bing Yan, Jianying Zhou

**Affiliations:** a Department of Respiratory Medicine; b Department of Pathology, First Affiliated Hospital, School of Medicine, Zhejiang University, Hangzhou, China.

**Keywords:** enzyme-linked immunosorbent assay, eosinophilia, pleuritis, sparganosis

## Abstract

**Rationale:**

Sparganosis is an infectious disease caused by a larval tapeworm of the genus *Spirometra*, which commonly invades subcutaneous tissues. Pulmonary and pleural involvement due to sparganum has been rarely reported previously.

**Patient concerns:**

We herein described a case of recurrent eosinophilic pleuritis in a 24-year-old woman. She was admitted with persistent cough and shortness of breath for more than 1 month. Initial chest computed tomography scan suggested right pleural effusion and diffuse pleural thickening. Slightly elevated eosinophil counts were found in both the peripheral blood and pleural fluid. She underwent right pleurectomy but histological examination failed to obtain an etiological diagnosis. Moreover, eosinophilic pleural effusion re-appeared in the contralateral thoracic cavity one month later. After re-admission, we reviewed her medical history meticulously and found she had a history of ingesting raw snake gallbladders before hospitalization. The final diagnosis was confirmed by the markedly positive reaction against sparganum antigen in both serum and pleural fluid sample.

**Diagnosis:**

Eosinophilic pleuritis caused by sparganum infection.

**Interventions:**

After the diagnosis, the patient was treated with praziquantel at 75 mg/kg/d for 3 days.

**Outcomes:**

Pleural effusion absorbed completely and eosinophil count in peripheral blood returned to normal range. No evidence of recurrent pleural effusion had been observed in over one year of follow-up.

**Lessons:**

Clinicians need to be aware the possibility of sparganum infection in cases of eosinophilic pleuritis. The specific enzyme-linked immunosorbent assay remains a useful method in acquiring a rapid diagnosis, especially when histological examination is unable to detect the larvae in the thoracic cavity.

## 1 Introduction

Human sparganosis is a rare parasitic infection caused by migrating tapeworm larvae of the genus *Spirometra*, which is distributed worldwide, especially in East Asia such as Korea and China.^[1,2]^ It is usually parasitic in cyclops as the first intermediate host, and second intermediate hosts are freshwater fish, amphibians, and reptiles. Wild carnivores are regarded as definitive hosts. Once infected, the plerocercoids are prone to migrate to soft subcutaneous tissues or muscles appearing as subcutaneous nodules with an obvious elevated level of eosinophils in the peripheral blood (Pb). Other parasitic infection sites including the eyes, brain, and the spinal cord also have been reported previously.^[3]^

The thoracic cavity is a rare site for the localization of this parasite. Here, we report a case of sparganosis, presenting as recurrent eosinophilic pleuritis without a subcutaneous mass or lump. Furthermore, we review the cases of pleural sparganosis reported in the PubMed database and analyze the common risk factors, clinical features, treatments and outcomes. This information would be helpful for clinicians to make an accurate diagnosis when managing eosinophilic pleuritis.

## 2 Case report

A 24-year-old Chinese woman was admitted to our respiratory department complaining of persistent cough and shortness of breath for more than one month. She had taken some antibiotics prescribed by her local clinic but without efficiency. Her past and familial medical histories were unremarkable. She had no history of cigarette smoking or alcohol drinking habit.

**Figure 1:**
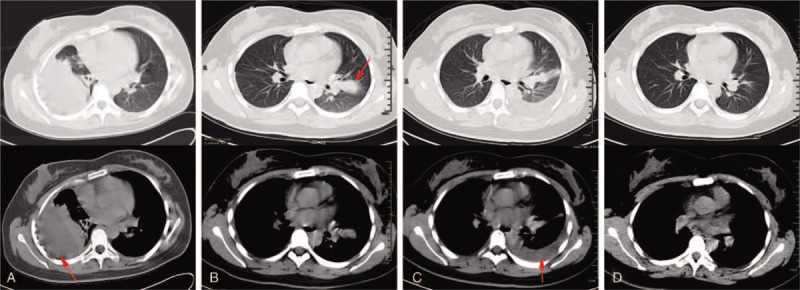
Chest CT performed during treatment. (A) Chest CT performed at first admission revealed right pleural effusion, pulmonary atelectasis, diffuse pleural thickening (red arrow) and decreased volume of right thoracic cavity. (B) One month after right pleurectomy, chest CT showed a new patch shadow (red arrow) in the upper lobe of left lung. (C) After giving an empirical antibiotic therapy, pulmonary shadow still existed, moreover, massive pleural effusion (red arrow) appeared in the left pleural cavity. (D) Pleural effusion and pulmonary shadow completely absorbed after praziquantel therapy. CT: computed tomography.

**Figure 2:**
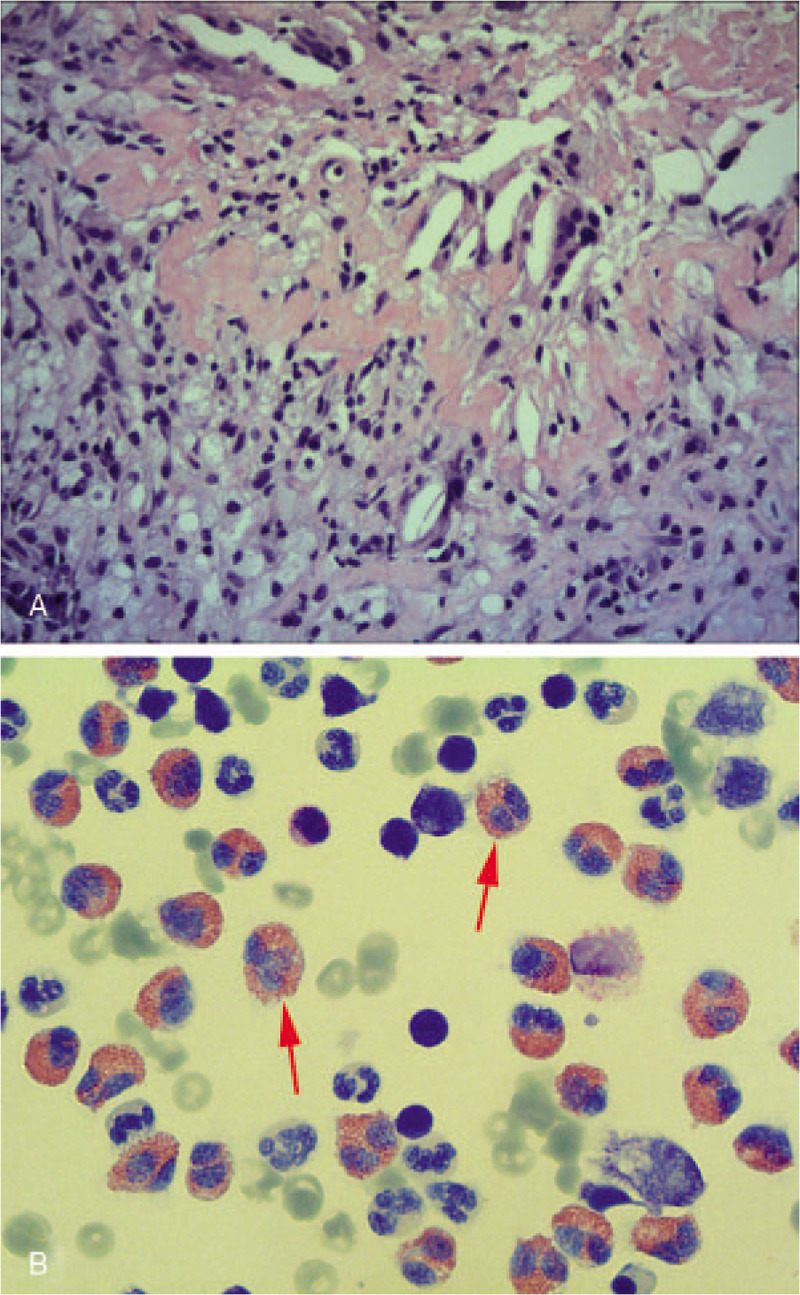
Pathological and cytological examinations performed during treatment. (A) Pathological test of the right pleura showed plenty of necrotic tissue, inflammatory cells and multinucleated giant cells, but no evidence of tapeworm was revealed in this histological image (Hematoxylin and eosin stain, × 200). (B) Increased number of eosinophils (red arrows) in the pleural fluid, obtained during her second admission, were observed under a microscope (Wright stain, ×1000).

On examination, her body temperature (37.2°C) and blood pressure (112/74 mm Hg) were normal. She had no conjunctival anemia, jaundice, superficial lymph node swelling, or subcutaneous mass. Cardiac sounds were clear. Breathing sounds were reduced in the right lung field. She had no abdominal hepatosplenomegaly or edema of either lower limb. Initial laboratory test results were presented in Table 1, including: white blood cell count of 5700/μL with 8.5% eosinophils, C-reactive protein: 7.6 mg/L. Other laboratory data such as blood biochemistries, tumor markers were within normal range. Chest computed tomography (CT) demonstrated right pleural effusion accompanied by pulmonary atelectasis, diffuse pleural thickening and decreased volume of right thoracic cavity (Fig. 1A). Diagnostic thoracocentesis was performed to obtain a fluid sample. The general appearance of the pleural fluid (Pf) was turbid, yellowish in color, contained numerous inflammatory cells (65% neutrophils, 25% lymphocytes, and 10% eosinophils). Levels of protein and lactate dehydrogenase were 53 g/L and 685 IU/L respectively. Adenosine deaminase (14 IU/L) and carcinoembryonic antigen (0.6 ng/mL) were within normal range. Malignant cells and mycobacterium tuberculosis were not detected. Cultures of bacteria were negative. A diagnosis of chronic empyema was suspected based on exudative pleural effusion and radiographic findings. Then, video-assisted pleurectomy was performed in an effort to obtain an etiological diagnosis and allow the lung to re-expand. As a result, pathological test of the pleura showed plenty of necrotic tissue, inflammatory cells and multinucleated giant cells, with a negative acid-fast stain (Fig. 2A). Considering no specific pathogen was detected, we advised a close clinical observation after surgery.

**Table 1:**
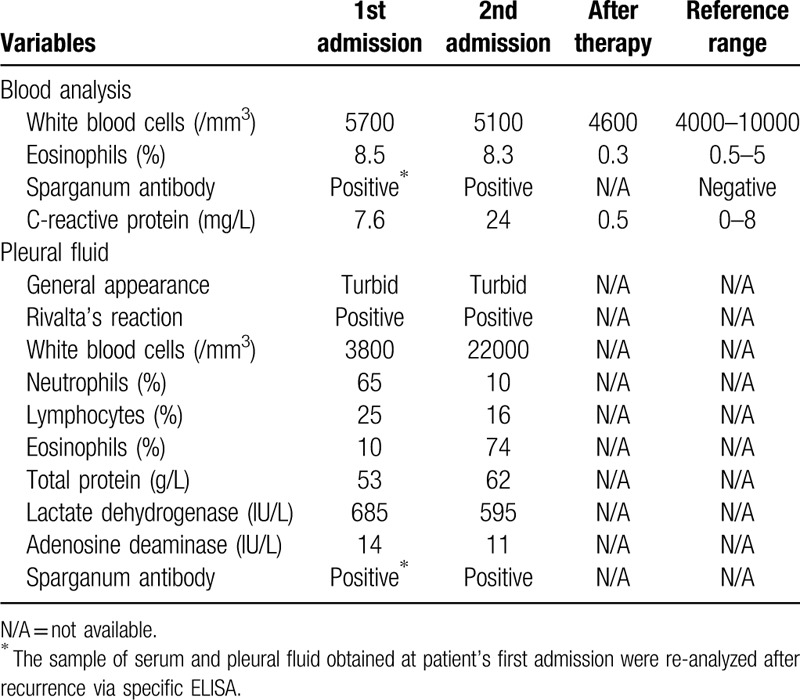
Patient's laboratory data.

One month after discharge, this patient returned to our outpatient clinic for a severe cough accompanied by occasional bloody sputum. She was febrile at 38.0°C. Chest CT revealed a new patch shadow located in the upper lobe of left lung while the right lung was almost normal (Fig. 1B). C-reactive protein was 24.6 mg/L. We prescribed moxifloxacin as an empirical antibiotic therapy for one week. However, her respiratory symptoms got worsened with the appearance of chest tightness. Her body temperature fluctuated between 37.5°C to 38.5°C. Chest CT was performed again and showed massive pleural effusion in left thoracic cavity and the pulmonary shadow still existed (Fig. 1C). Thoracentesis was carried out to make a definitive diagnosis. The Pf was turbid, yellow in color, containing 22000/μL of granulocytes and eosinophils accounted for 74%. Protein, adenosine deaminase and lactate dehydrogenase were 62 g/L, 11 IU/L, and 595 IU/L respectively. Meanwhile, 8.3% eosinophils with a white blood cell count of 5100/μL was detected in Pb (Table 1). We still failed to detect mycobacterium tuberculosis, ordinary bacteria, and malignant cells in Pf.

Considering the significantly elevated eosinophil counts in both the Pb and Pf, we suspected parasitic infection. Then, we reviewed her medical history meticulously and found that she had a history of ingesting raw snake gallbladders for several times three months before hospitalization in order to improve eyesight. Snake gallbladder, a traditional Chinese medicine, had been used to improve visual acuity and relieve arthritic pain for more than thousand years. But raw snake gallbladder may contain potentially pathogenic microbes and parasites. Hence, her serum and Pf were examined by enzyme-linked immunosorbent assay (ELISA) to detect specific antibody against various parasitic antigens including: *Toxocara canis, Ascaris suum, Paragonimus westermanii, Cysticercus cellulosae, Fasciola hepatica, Sprirometra erinaceieuropaei plerocercoid,* et al. As a result, both the sample of serum and Pf revealed a markedly positive reaction against sparganum antigen without cross-reactions with other parasitic antigens. Moreover, the samples of serum and right Pf which obtained and preserved in laboratory during her first hospitalization, were re-analyzed and also revealed positive results. Finally, we made the diagnosis of pleural sparganosis according to the high-risk behavior, clinical features, especially the positive reaction against sparganum antigen in blood and pleural effusion test. Then, the patient was treated with praziquantel at 75 mg/kg/d for 3 days. Symptoms such as fever, cough and hemoptysis were improved soon after therapy. Four weeks later, left pleural effusion and pulmonary shadow absorbed completely (Fig. 1D), eosinophil count in Pb returned to normal range as well (Table 1). Furthermore, no evidence of recurrence had been observed in over 1 year of follow-up.

## 3 Discussion

Though human sparganosis is distributed worldwide, most of the cases occur in East Asia. From 1959 to 2012, more than 1000 valid cases of human sparganosis had been reported in mainland China.^[3]^ The relatively higher prevalence rate in these countries is associated with unusual habits of eating uncooked frog and snake flesh, drinking stream water or application of frog meat as a poultice. In current case, we speculated that the patient was infected by this worm via ingesting raw snake gallbladders. The worm may penetrate intestinal wall and diaphragm, finally causing infection in the thoracic cavity.

Vast majority of human sparganosis appears as slow growing and migratory subcutaneous nodules in muscles or subcutaneous tissues. Visceral sparganosis (lungs, intestine and urogenital system) is relatively infrequent. Kim et al. reported 438 Korean sparganosis cases and showed the involvement of visceral organs was around 7.8%.^[14]^ The incidence in China was similar (7.1%), according to the research by Lu et al.^[3]^ Thoracic cavity is a rare site for the localization of this parasite. To the best of our knowledge, only a few cases presented as pleural effusion had been reported around the world.^[4–10]^ We identified seven reported cases of pleural sparganosis in the PubMed database and summarized clinical characteristics in Table 2. The median age of these previous cases was 46 years (range 27 - 62 years). The most common high-risk behaviors for infection were raw flesh ingestion (N = 4, 57.1%) and drinking un-boiled water (N = 2, 28.6%). The symptoms were non-specific, including fever, cough, dyspnea, and pleuritic chest pain, et al. Lymphodominant exudative pleural effusions with eosinophilia (range 10% - 78%) and peripheral eosinophilia (range 6.2% - 26.9%) might be relatively common clinical features. Due to lack of specific symptoms and physical signs, pleural sparganosis was easily misdiagnosed as bacterial or tuberculous infection. A detailed life history, especially with high-risk behaviors, was an important clue for differential diagnosis.

**Table 2:**
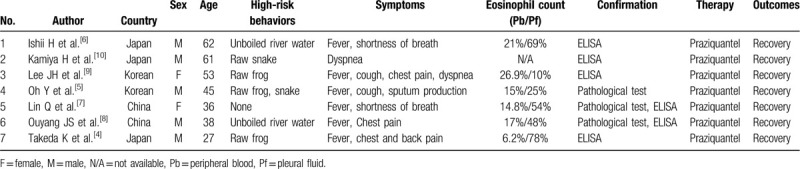
Clinical characteristics of pleural sparganosis.

Usually, the confirmation of human sparganosis is established mainly by pathological detection of worms. Lin et al. reported plerocercoid of the *S. erinacei-europaei* migrating along the parietal pleura found by thoracoscopy.^[7]^ While, Takeda et al. described a similar case of acute empyema caused by sparganum mansoni but failed to confirm by thoracoscopic examination.^[4]^ Actually, it could not be always successful to detect the larvae in the thoracic cavity for various reasons, for example, the migration of worm to lung, the visible range limitation of thoracoscopy, or inability to recognize the appearance of parasite by clinicians. Of all the eight pleural sparganosis cases (including present case), sparganosis bodies were confirmed by pleural histological findings in three cases (37.5%), while serum sparganum antibody assays were strongly positive in seven cases (87.5%). It indicated that an immunoserologic test remained to be critical for the diagnosis. The presence of anti-sparganum antibody in serum measured by ELISA, was known to have up to 90% sensitivity and specificity in confirmation according to previous research.^[11]^ Besides Pb, specific ELISA of the other body fluid, such as Pf, bronchoalveolar lavage fluid, pericardial effusion, cerebrospinal fluid, also could be considered as an effective diagnostic tool for sparganosis.^[4,12,13]^ Nevertheless, the method of ELISA still has some limitations such as its inability to distinguish current disease from previous infections. Thus, when patient with eosinophilic pleuritis possessed a positive ELISA but an absence of pathological result, further clinical assessments including epidemiology, high-risk behaviors and newly developed eosinophilia are necessary in the achievement of final confirmation.

To date, surgical removal remains a principle treatment of human sparganosis, but it cannot be applied in patients with pleural effusion, multiple tiny lesions, or an ongoing insidious infection. In current case, though patient underwent pleurectomy, pulmonary infiltration and eosinophilic pleural effusion re-appeared in contralateral thoracic cavity soon after surgery. We considered that the recurrence was strongly related to the migration of worm among different visceral organs after incomplete surgical resection. The living worms disseminated to distance through the airway or loose connective tissue, resulting in a new infection in contralateral lung. Concerning the chemotherapy, praziquantel should be an alternative treatment in surgically unresectable case. It had been proved effective for pleural sparganosis according to previous literatures and no evidences of recurrence were reported by far.^[4–10]^ It was also confirmed by the successful treatment with 3 consecutive doses of praziquantel (75 mg/kg/d) in this case.

## 4 Conclusion

We report a rare case of recurrent eosinophilic pleuritis caused by sparganum infection. It indicates that a specific ELISA is very useful in differential diagnosis for eosinophilic pleuritis and praziquantel is an effective medicine for the treatment of sparganum infection. Moreover, we consider that health education to avoid ingestion of raw frogs, snakes and un-boiled water is fairly important for the prevention of parasitic infections.

## Author contributions

**Conceptualization:** Liangjie Fang, Yuehong Wang, Jianying Zhou.

**Data curation:** Liangjie Fang.

**Investigation:** Liangjie Fang, Yuehong Wang, Jianjing Zhou.

**Methodology:** Qiqi Gao, Bing Yan.

**Writing – original draft:** Liangjie Fang.

**Writing – review & editing:** Liangjie Fang, Yuehong Wang, Jianying Zhou.
